# Acute renal failure during immediate post transplant period due to a pericardial effusion

**DOI:** 10.1186/s13104-015-1571-4

**Published:** 2015-10-20

**Authors:** Ranga Migara Weerakkody, Pushpa Nandani Lokuliyana, Mohammed Hussain Rezvi Sheriff

**Affiliations:** University Medical Unit, National Hospital of Sri Lanka, Regent Street, Colombo 9, Sri Lanka; Department of Anesthesiology, National Hospital of Sri Lanka, Regent Street, Colombo 9, Sri Lanka; Department of Clinical Medicine, Faculty of Medicine, University of Colombo, Kynsey Road, Colombo 8, Sri Lanka

**Keywords:** Renal transplant, Pericardial effusion, Doppler sonography, Delayed graft function, Hypoalbuminemia

## Abstract

**Background:**

Pericardial effusions and acute renal failure are common findings in clinical practice. However, acute renal failure resulting from pericardial effusions (without tamponade) is a rare finding. We report the first such case to occur in a transplanted kidney.

**Case presentation:**

A 20-year-old Sri Lankan male presented with hypertensive crisis in the background of end stage renal failure. He was thoroughly investigated for secondary causes of hypertension to no avail. He was hemodialysed adequately for 6 months, while being worked up for transplantation. He received an ABO matched, living donor transplant. Immediate post-operative period his urine outputs were poor, soon to became anuric by 6 h post-transplant. Elevated liver enzymes and non-specific increase of resistivity indexes (0.84–0.88) at the Doppler scan raised the possibility of venous hypertension. An echocardiogram showed a moderately large pericardial effusion which was tapped, and found to be a transudate. He started producing urine within 6 h, entered polyuric phase by day 3, and by day 7 his creatinine dropped to reference levels. Vasculitis screen, anti nuclear factor, viral screen, and rickettsia serology were negative. Albumin levels on day 2 were 27 g/l and were replaced using human albumin. The exact cause of pericardial effusion is unclear but hypoalbuminemia, drug-induced and idiopathic are possible causes. He has excellent graft function, no recurrences or constrictive pericarditis after 2 years follow.

**Conclusion:**

We recommend any patient who has delayed graft function and raised central venous pressures to have an echocardiogram to exclude pericardial effusions. The response to pericardiocentesis had been universally good in reported cases.

## Background

Pericardial effusions and acute renal failure (ARF) are common findings in clinical practice. Acute renal failure caused by cardiac tamponade, is well known phenomenon [1–4]. However pericardial effusions which are not severe enough to cause tamponade, presenting as ARF is rare [5]. Only six cases have been reported [3–8], and native kidneys were affected in all of them. We report the first case to occur in a transplanted kidney.

## Case presentation

A 20 year old Sri Lankan male, presented with blood pressures of 270/140 mmHg and fits. We managed his condition with antihypertensives, antiepileptics and diagnosed posterior reversible encephalopathy syndrome. He was uremic, had a serum creatinine of 1250 µmol/l, proteinuria of 200 mg/dl, contracted kidneys in ultrasonography and was anemic, supporting a diagnosis of end stage renal failure (ESRF). We could not obtain a tissue diagnosis for the primary renal pathology as contracted kidneys, were un-biopsiable. He had a 24 h protein excretion of 2.2 g/day, and had urine output of 500–600 ml/day. Serum markers of vasculitis - Antinuclear factor, rheumatoid factor, complement levels (C_3_ and C_4_), anti neutrophilic cytoplasmic antigens (ANCA), hepatitis profile and Human immuno deficiency virus (HIV) - were negative. We excluded causes of secondary hypertension such as pheochromocytoma and Conn’s disease with biochemical tests (electrolytes, aldosterone levels and urine meta-nephrines) and imaging (MRI). His mother aged 48, was worked up as a donor for transplant. Pre-transplant cardiac assessment of the recipient showed left ventricular hypertrophy, and grade 2 diastolic dysfunction. He was hemodialysed three times weekly for 6 months (Kt/V > 1.4) and became anuric 3 months after initiating renal replacement therapy. He and his donor were identical to the status Cytomegalovirus (CMV) and Epstein Barr Virus (EBV) IgG, both being positive. Patient was from upper-middle class family and had excellent hygienic practices and nutritional status.

**Fig. 1:**
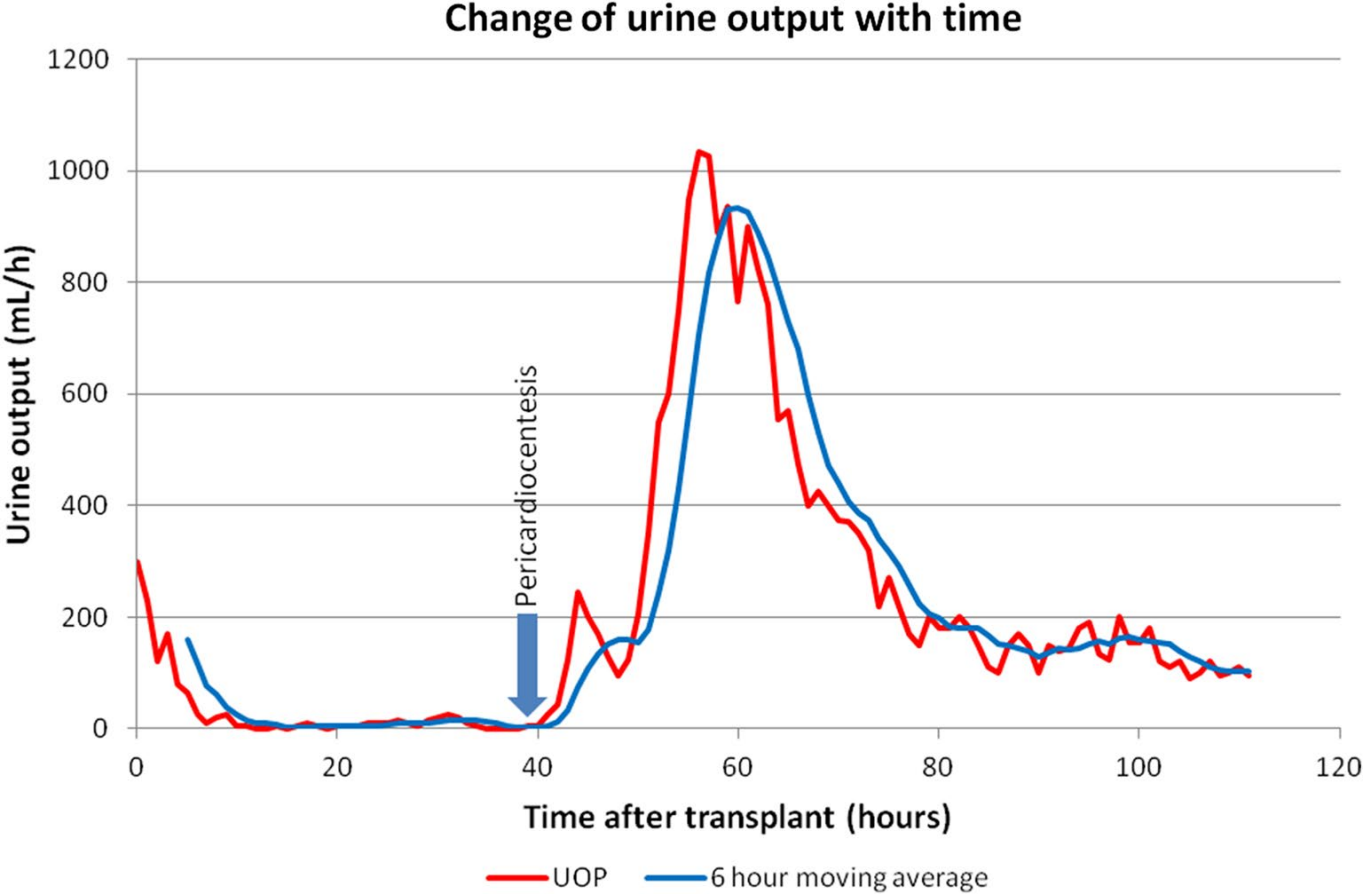
Changes of the urine output in relation to time and response to pericardial aspiration

He underwent renal transplantation on June 2011, induced with local protocol of single dose of basilixumab (20 mg), cyclosporin (CyA) 12.5 mg/kg/day, mycophenolate mofetil 30 mg/kg/day and prednisolone 0.5 mg/kg/day. Cold ischemia time was 144 min, and warm ischemia time was 22 min. Additionally, he was on glyceryl tri-nitrate infusion for control of blood pressure in first 24 h, converted to amlodipine 5 mg od, bisoprolol 5 mg bd and prazocin 2 mg tds. The immediate post-transplant period was complicated with oliguria and rising serum creatinine. He had a urine output of 300 ml/h in first hour, which dwindled gradually, to become anuric in 6 h’ time. We noted that blood pressures were within target levels, (systolic 140–160, diastolic 80–90 mmHg), and mild increase of heart rate (95–105/min). Central venous pressures (CVP) has rose from 12 to 25 cm of water, with no evidence of pulmonary edema. Due to increasing CVPs and creatinine levels from 430 to 850 μmol/l, he was dialyzed on post transplant day 1. Ultrasonography and duplex showed no hematoma, vascular compression or obstruction, and a resistivity index (RI) of 0.84–0.88, slightly high for a first day of transplantation. We performed a renal biopsy and CyA levels on day 2 in anticipation of acute rejection since he was not improving. While the patient was waiting on the biopsy reports, we noticed liver function tests has elevated more than three times [from alanine transaminase (ALT) 11 to 97, aspartate transaminase (AST) 9 to 77], with a serum albumin level of 27 g/l. This led us to exploration of causes for combined liver and renal ischemia. Echocardiography revealed a large pericardial effusion without features of tamponade [no diastolic collapse of right ventricle, IVC dilatation present (2.1 cm) but no loss of respiratory variations, no respiratory increase of inter-ventricular dependence, respiratory variations <25 % in mitral, aortic and/or tricuspid flow on doppler]. Emergency pericardial drainage was performed, with 650 ml of serous fluid aspirated, ultimately proved to be a transudate (see Table 1). We started patient on daily 20 % human albumin solution 100 ml. We did not consider non steroidal anti-inflammatory medications because of renal toxicity, use of steroids, and being free of symptoms of pericarditis. Within 6 h of drainage patient started producing urine, and entered the polyuric phase of post transplantation, by day 3. CMV, EBV, coksackie and rickettsia serology was negative. Thyroid stimulating hormone (TSH) levels of 2.4 U and blood cultures were sterile. Post transplant ANA or complement levels were not performed. His creatinine dropped rapidly and reached a plateau of 110 μmol/l by day 7. CyA trough levels were 233 ng/ml which was consistent with mild toxicity. Renal biopsy showed no abnormalities apart from mild acute tubular necrosis, which was expected for the timing of the biopsy and ischemia times of the allograft. We diagnosed pre-renal acute renal failure due to pericardial effusion. He was followed up with weekly echocardiograms for 1 month and then monthly for 6 months, and did not show recurrence or signs of constrictive pericarditis. Two years into his transplant, he is well with excellent graft function (S. Creatinine 122 μmol/l) and a quality of life. Figure 1 shows changes of urine output with relation to post transplant time and time following pericardiocentesis. Table 2 shows changes of biochemical and imaging parameters during the hospital stay.

**Table 1:** Pericardial fluid analysis

Parameter	Value	Comments
Appearance	Clear	
Specific gravity	1.011	
pH	7.4	
Proteins	12 g/l	Pericardial fluid: serum proteins ratio = 0.44
Glucose	5.2 mmol/l	Random blood glucose = 6.2 mmol/l
Fluid LDH: serum LDH	0.31	
Cells	None	

*LDH* lactate dehydrogenase

**Table 2:** Trends of results of blood investigations and the resistivity indexes over the first seven days of transplant

	Duration after surgery												
	Pre-op	1		2		3		4		5		6	7
		M	E	M	E	M	E	M	E	M	E		
SCr (mmol/l)	396	430	490	850	413	632	432	393	335	228	170	142	117
K^+^ (mmol/l)	5.3	5.6	5.6	6.1	4.0	5.2	4.4	5.5	4.2	3.6	3.4	3.5	3.9
Na^+^ (mmol/l)	142	144	140	139	141	141	139	142	146	147	147	144	141
ALT (IU)	11	12		32		97		86					24
AST (IU)	7	7		19		77		74					13
Albumin (g/l)	33	31		27		36		39					44
Uric acid (mg/dl)	5.8	5.6											4.5
RI		0.86		0.87		0.75		0.73		0.70			0.66

*ALT* alanine transaminase, *AST* aspartate transaminase, *SCr* serum creatinine, *RI* resistivity index, *M* morning, *E* evening

## Discussion

Acute renal failure and pericardial effusions are common conditions. Interestingly it is very rare for a pericardial effusion to present as ARF. Only six such cases have been described in the literature [3–8], and all of them involved native kidneys. Allograft dysfunction results with increased venous pressure and reduced cardiac output, following constrictive pericarditis [9, 10], to best of our knowledge this is the first case, where a pericardial effusion is involved instead of constrictive pericarditis. Our patient had dramatic improvement following decompression, similar to previously reported cases with native kidneys. The pathophysiology of the ARF is hypothesized as decrease of transmural pressure and cardiac output resulting in renal ischemia, reducing glomerular filtration rate (GFR) [11]. In comparison to a native kidney, an allograft is supplied by a small caliber vessel, and receives a lesser perfusion pressure. A relatively modest drop of blood pressure can bring about devastating effects on the graft. The graft is extremely vulnerable to effects of hypotension due to de-nervation and limited capacity on auto-regulation [12]. Pericardial effusions are associated with high right atrial and caval pressures, and allografts are known to readily develop ischemia under such circumstances. This patient had high caval pressures (CVP of 25 cm) impeding the allograft blood flow. The reduction in stroke volume manifest itself as a low RI in the graft. It may impede the arterial flow and mimic renal vein thrombosis (RVT). Resistivity Indices are >1.0 in complete RVT and 0.8  to  1.0 in partial RVT [13]. The normal pulse pressure and satisfactory blood pressures rule out reduction of stroke volume, and venous hypertension is the most likely cause for the renal ischemia. Overlooking of rising CVP delayed the diagnosis, which was unfortunately attributed to fluid overload. The diagnosis became more challenging  because of muted response of tachycardia,  (beta blockers) and non-specific RIs.

Etiologies of previously reported cases were suppurative, neoplastic or idiopathic [5]. Commonest etiologies are viral and connective tissue disorders. A negative viral screen, and pre-transplant negative vasculitis and connective tissue disorders screen make above etiologies less possible. Unknown underlying nephropathy with systemic manifestations is also a possibility, especially given that we had no tissue diagnosis for the primary nephropathy. But lack of recurrence makes this less likely diagnosis. Hypoalbuminemia was the only modifiable factor in our patient, and dramatic response to albumin indicates the etiology of the effusion. However, drug induced and idiopathic causes for the effusion cannot be excluded, and remain as possible etiologies. The traditional definition of delayed graft function (DGF) rests on dialysis requirement during the first week after transplantation. A more functional definition of DGF is defined as a failure of the serum creatinine to decrease by at least 10 % daily on 3 successive days during the first week [14]. DGF could only be diagnosed in the absence of factors contributing to pre-renal, renal or post renal acute kidney injuries, making pericardial effusions an unlikely cause for DGF.

## Conclusion

Pericardial effusions can accumulate without much notice during post transplant period and can cause acute renal failure. Monitoring CVP and Echocardiography are very helpful in establishing the diagnosis. The Doppler sonography usually is helpful, but peculiar hemodynamic conditions can lead to non-specific results. The response to pericardiocentesis had been universally good in reported cases.

## Consent

Written informed consent was obtained from the patient for publication of this Case Report.

## Abbreviations

ALT: Alanine aminotransferease
ANA: Antinuclear antibodies
ARF: Acute renal failure
AST: Aspartate aminotransferase
CMV: Cytomegalovirus
CVP: Central venous pressure
CyA: Ciclosporin
DGF: Delayed graft function
EBV: Epstein Barr virus
GFR: Glomerular filtration rate
IVC: Inferior vena cava
RI: Resistivity index
TSH: Thyroid stimulating hormone
RVT: Renal vein thrombosis
